# A New Hope in Spinal Degenerative Diseases: Piezo1

**DOI:** 10.1155/2021/6645193

**Published:** 2021-01-25

**Authors:** Daxue Zhu, Guangzhi Zhang, Xudong Guo, Yidian Wang, Mingqiang Liu, Xuewen Kang

**Affiliations:** ^1^Lanzhou University Second Hospital, 82 Cuiyingmen, Lanzhou 730030, China; ^2^Orthopaedics Key Laboratory of Gansu Province, Lanzhou 730030, China

## Abstract

As a newly discovered mechanosensitive ion channel protein, the piezo1 protein participates in the transmission of mechanical signals on the cell membrane and plays a vital role in mammalian biomechanics. Piezo1 has attracted widespread attention since it was discovered in 2010. In recent years, studies on piezo1 have gradually increased and deepened. In addition to the discovery that piezo1 is expressed in the respiratory, cardiovascular, gastrointestinal, and urinary systems, it is also stably expressed in cells such as mesenchymal stem cells (MSCs), osteoblasts, osteoclasts, chondrocytes, and nucleus pulposus cells that constitute vertebral bodies and intervertebral discs. They can all receive external mechanical stimulation through the piezo1 protein channel to affect cell proliferation, differentiation, migration, and apoptosis to promote the occurrence and development of lumbar degenerative diseases. Through reviewing the relevant literature of piezo1 in the abovementioned cells, this paper discusses the effect of piezo1 protein expression under mechanical stress stimuli on spinal degenerative disease, providing the molecular basis for the pathological mechanism of spinal degenerative disease and also a new basis, ideas, and methods for the prevention and treatment of this degenerative disease.

## 1. Introduction

Piezo1 is a mechanically sensitive ion channel protein that was newly discovered by Coste et al. [1] in 2010. The main function of piezo1 is to sense, conduct, and convert mechanical signals on the cell membrane, and it plays a vital role in mechanics among humans and other mammals. In recent years, studies on piezo1 have gradually increased and deepened. The piezo1 protein has been found to be stably expressed stably not only in the respiratory, cardiovascular, gastrointestinal, and urinary systems [2] but also in human mesenchymal stem cells (MSCs), osteoblasts, osteoclasts, chondrocytes, and nucleus pulposus cells. These cells can all receive external mechanical stimulation through the piezo1 protein channel to affect their proliferation, differentiation, migration, and apoptosis.

### 1.1. Mechanism of Spinal Degenerative Diseases

Spinal degenerative diseases include diseases involving the degeneration of the bony vertebrae and intervertebral discs [3]. Clinically, most low back pain occurs due to degenerative changes in the nucleus pulposus of the intervertebral disc [4–6]. The increase in osteoclasts leads an increased osteoclast effect, decreased MSCs lead to decreased osteoblast differentiation, and decreased osteoblasts themselves can affect the bone mass and density of the vertebral body, which are also important factors leading to osteoporosis and osteoporotic fractures [7–9]. The intervertebral disc is the soft connective tissue that connects the adjacent vertebral bodies of the spine. It is a complex tissue composed of the nucleus pulposus, annulus fibrosus, and cartilage endplates [10, 11]. The intervertebral disc has the function of transmitting and buffering spinal stress caused by body weight and muscle contraction. The nucleus is a gel-like substance composed of nucleus pulposus cells and mainly acts to resist the longitudinal pressure transmitted up and down the spine and absorb shock. The annulus fibrosus is rich in cross-arranged type I collagen fibers and annular fibroblasts, and its main function is to cushion the lateral expansion of the intervertebral disc [12, 13]. Their degeneration can be manifested as nucleus pulposus cell apoptosis and rupture of the annulus fibrosus cells, which lead to narrowing of the intervertebral space and a herniated nucleus pulposus compressing the nerve root or spinal cord. The cartilage endplate is composed of hyaline cartilage matrix and endplate chondrocytes [14]. It mainly connects the intervertebral disc with the adjacent vertebral body and provides nutrition for the intervertebral disc as a metabolic channel [15]. Degeneration of the cartilage endplate can be expressed as endplate inflammation, calcification, etc. Spinal degenerative disease is a common clinical disease, and initial degeneration of the intervertebral disc may appear in adolescence, as many as 20% of young people have mild symptoms [16]. The incidence of spinal degenerative disease increases with age. Approximately 10% of 50-year-old men suffer from this disease, and 50% of 70-year-old men have this disease [17, 18]. In some reports, degenerative disease of the intervertebral disc is present in 90% of people; many of them have no signs of the disease [19, 20]. In response to mechanical stress stimuli, piezo1 is expressed in all of the abovementioned cells. Piezo1 affects the density and intensity of the vertebral body and the disc tissue, functioning by affecting cellular differentiation, proliferation, or apoptosis; thus, it is an indirect factor associated with the occurrence and development of spinal degenerative diseases. This article reviews the latest studies on the mechanism of action of piezo1 in the vertebral body and intervertebral disc-related cells, summarizes the latest research progress, and systematically explains the role of piezo1 in spinal degeneration to find new molecular targets for spinal degenerative diseases and provide new ideas and methods for treatment.

### 1.2. Piezo1

**Figure 1:**
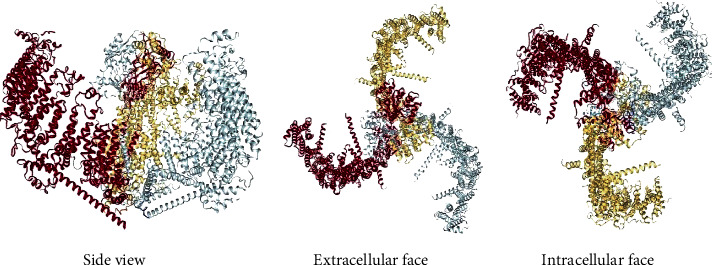
Structure of piezo1 with the cryoelectron microscopy [32–35].

Piezo1, a mechanosensitive ion channel protein, was first discovered in a mouse neuroblastoma cell line in 2010 by the Patapoutian team of the Scripps Research Institute. It is a large protein with more than 2,000 residues that crosses the cell membrane approximately 30 to 40 times; piezo1 is located on chromosome 16, which is encoded by the Fam38A gene, and has a molecular weight of approximately 320 kDa [1, 21]. It is composed of different separable modules, which coordinate the sensing and transduction of mechanical stimulation by conducting ions. In addition, this protein channel is also a mechanically sensitive ion channel that depolarizes to the nonselective permeation of cations [22]. The piezo1 protein channel is permeable to Na^+^, K^+^, Ca^2+^, and Mg^2+^, but it is more permeable to Ca^2+^ than to other positive ions [23, 24]. Furthermore, experiments have shown that signal transduction via the piezo1 protein channel occurs through Ca^2+^. Ca^2+^ acts as a second messenger in the signal transduction pathway [25]. In addition, it is also a low-threshold (1-3 mN/m), fast-inactivated, and small-conductance protein channel [26]. However, its spatial structure was not discovered until 2017, when researchers revealed the overall structure of the piezo1 protein with cryoelectron microscopy (Figure 1): it has a propeller-like shape with three curved “blades” surrounding the central hole, and the top is covered by a cap called the C-terminus [27–31]. The central channel part is composed of approximately 350 amino acids at its carboxyl end, including an outer helix, extracellular C-terminal domain, inner helix, and intracellular C-terminal domain. Each spiral blade contains three main structural components including the “blade, beam, and anchor” [32–35].

This gives the piezo1 protein channel a unique 38-transmembrane-helix topology and designated mechanical sensor components, allowing it to have a lever-type mechanical mechanism [36–38]. When the piezo1 protein channel is activated, its peripheral leaves can be used as a lever-like device to perform effective long-distance allosteric gate control and respond to different forms of mechanical stimuli, such as poking and stretching [39–44], through conformational changes to achieve a chemically and mechanically gated lever transduction pathway [45–48].

**Figure 2:**
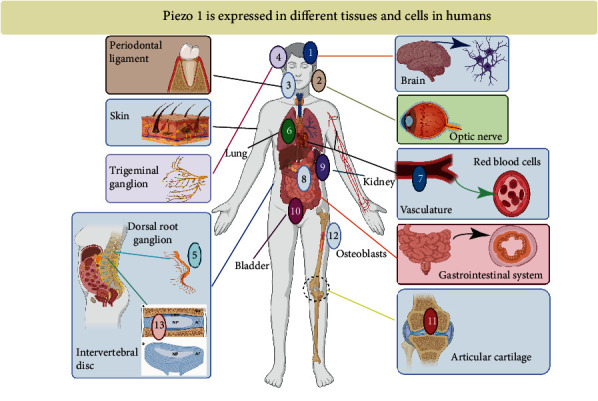
Piezo1 is expressed in different tissues and cells in humans (adapted from Reference [50]).

In addition, piezo1 is expressed in most mammals. Studies have confirmed that piezo1 is also widely expressed in various organs and tissues of the human body, such as the following: (1) brain, (2) optic nerve head, (3) periodontal ligament, (4) trigeminal ganglion, (5) dorsal root ganglion and skin, (6) lungs, (7) cardiovascular system and red blood cells, (8) gastrointestinal system, (9) kidneys, (10) bladder, (11) articular cartilage, (12) osteoblasts, and (13) nucleus pulposus cells (Figure 2) [2, 49]. The existing research also shows that more than 25 gene mutations in piezo1 are related to human diseases. For example, a mutated piezo1 protein channel allows excessive calcium ions to pass through, leading to the downstream activation of potassium channels. The subsequent outflow of potassium ions causes changes in intracellular osmotic pressure that dehydrates red blood cells and ultimately leads to hemolytic anemia [38, 50]. Therefore, due to the expression of piezo1 in a variety of human tissues and cells, its mutation or abnormal expression is inevitably closely related to a variety of human diseases, including spinal degeneration.

## 2. Role of Piezo1 in Spinal Degeneration

### 2.1. Piezo1 Regulates the Differentiation of Mesenchymal Stem Cells

Many studies have confirmed that bone marrow MSCs can differentiate into osteoblasts and bone marrow adipocytes [51, 52]. In elderly patients with spinal degeneration, the onset of osteoporosis, a common metabolic bone disease, is related to the destruction of bone metabolism [53]. The fundamental reason for the development of this disease is that the ability of MSCs to differentiate into osteoblasts is weakened while their ability to differentiate into adipocytes is enhanced, leading to increased bone marrow adipose tissue in the vertebral body and the loss of vertebral bone mass, reducing the bone density and hardness of the vertebral body [54, 55]. The differentiation of MSCs is influenced by many factors including cytoskeleton hardness, oxygen concentration, three-dimensional skeleton structure, and medium composition [56, 57]; however, the differentiation direction and self-renewal ability of MSCs are mainly affected by mechanical stress; thus, mechanical stress plays an irreplaceable role in the formation and growth of bone homeostasis [58, 59]. As a mechanically sensitive ion channel that has the function to sense, transform, and conduct signals of mechanical stress, the piezo1 protein channel directly or indirectly affects the degree of vertebral body degeneration by affecting the differentiation, migration, and apoptosis of MSCs [60].

**Figure 3:**
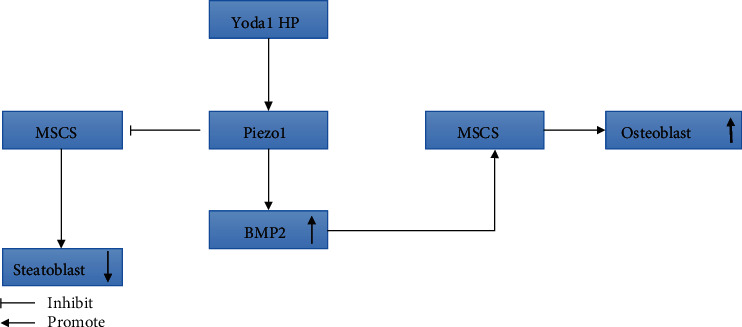
Piezo1 affects the differentiation of MSCs by regulating the expression of BMP2.

In the study of the piezo1 protein in MSCs, Sugimoto et al. [61] found that hydrostatic pressure promotes bone differentiation when studying its effect on the cell fate of MSCs depending on the expression of bone morphogenetic protein 2 (BMP2). BMP2 is an important growth factor for MSCs to differentiate into osteoblasts [62, 63], and when the piezo1 protein channel is activated, it can promote the expression of BMP2 in MSCs, facilitating their differentiation into osteoblasts while inhibiting their differentiation into adipocytes (Figure 3). In addition, they also used the piezo1 protein channel agonist Yoda1 to simulate the mechanical stimulation of the piezo1 protein channel. The results showed that Yoda1 can also induce BMP2 expression and promote osteoblast differentiation, while negatively regulating the differentiation of MSCs into adipocytes. This finding validates the previous experimental results and also shows that it is possible to control the differentiation direction of MSCs into osteoblasts or adipocytes by regulating signal transduction of the piezo1 protein channel without mechanical stimulation, making this channel the decisive factor in the fate of MSCs.

**Figure 4:**
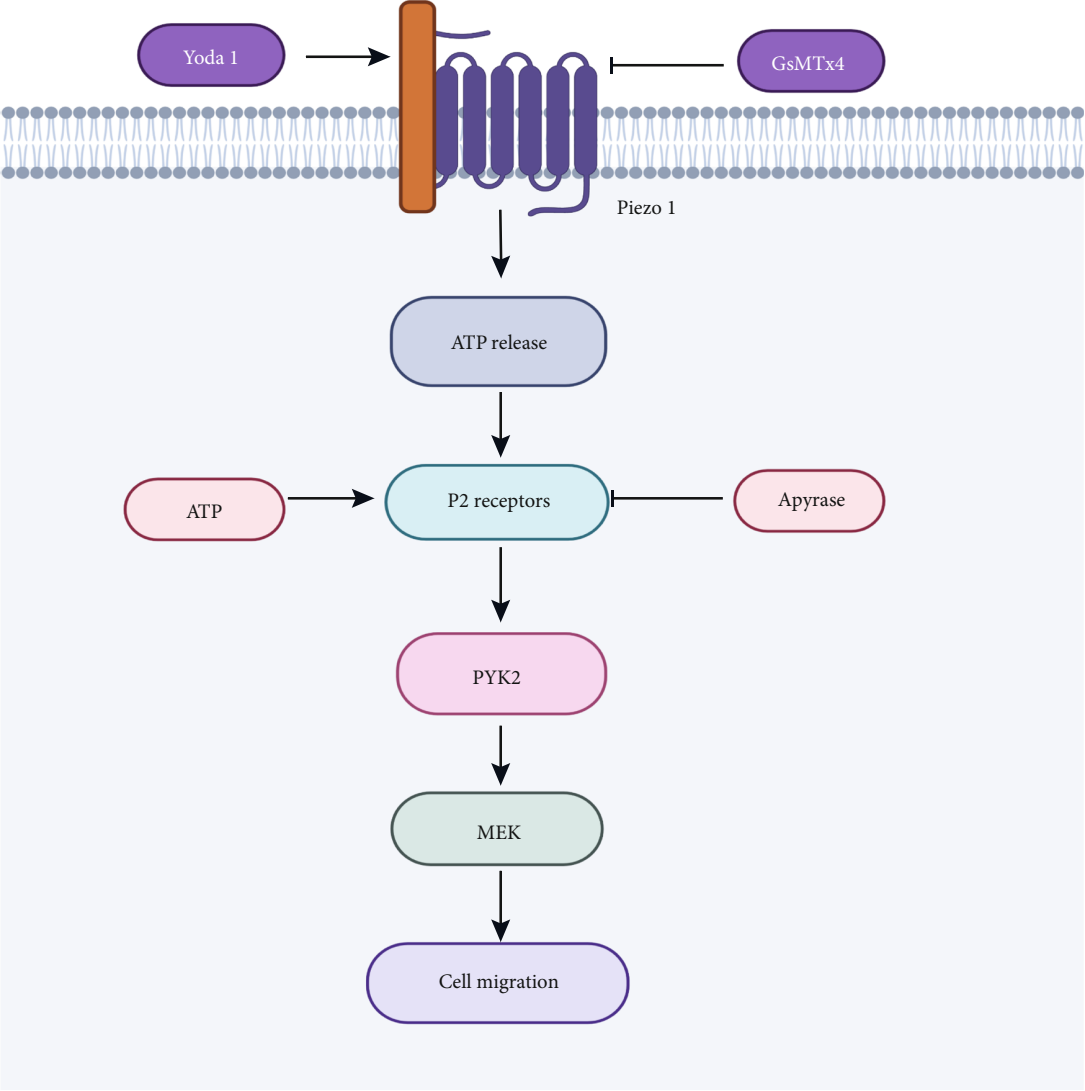
Mechanism of action of piezo1 in the migration and transformation of MSCs.

According to the timing of mechanical stretch stress, the researchers divided the MSCs into different groups and applied the inhibitor GsMTx4 [64] to study the effect of piezo1 on the transformation of MSCs at different times. The results also indicated that the mechanosensitive piezo1 ion channel can mediate the transformation of MSCs into other cells. However, although these experiments confirmed that piezo1 mediates the transformation of MSCs, the specific signaling pathway has not been reported. Until recently, the latest research [65] showed that activating the piezo1 protein channel can induce the release of ATP, which activates downstream signaling pathways PYK2 and MEK/ERK after the purinergic receptor P2 receives the signal to regulate the migration and transformation of MSCs (Figure 4) [66–69]. Therefore, piezo1 can affect the differentiation, proliferation, and metastasis of MSCs, destroy bone homeostasis, affect the hardness and density of vertebral bone, and participate in the occurrence of spinal degeneration.

### 2.2. Piezo1 Regulates Osteogenesis of Osteoblasts

Osteoblasts are the unit cells that remodel bones, accounting for 4–6% of all resident cells in bones [70]. In the traditional view, osteoblasts ultimately form bone cells that are essential for bone growth and maintenance [71]. However, studies now show that piezo1 can actively regulate the formation and function of osteoclasts and the homeostasis of hematopoietic stem cells [72]. It is also an endocrine cell that affects energy metabolism, male fertility, and cognitive ability by releasing osteocalcin [73, 74]. In the vertebral bodies of the elderly, osteoporosis caused by osteoblast dysfunction, which leads to weak bones and osteoporotic fractures, which are factors associated with spinal degeneration [75]. Modern drugs used to treat osteoporosis enhance the function of osteoblasts by changing their metabolism [76]. Therefore, these cells play an important role in spinal degeneration.

Researchers have found that piezo1 is expressed in osteoblasts and confirmed that it is involved in mediating mechanical reactions in bone and bone formation in mice [77]. Sugimoto et al. [61] reported that the activation of the piezo1 protein channel can not only induce the differentiation of MSCs but also induce the expression of BMP-2 through ERK1/2 and p38MAPK signaling. BMP-2 subsequently induces the expression of Runt 2 in osteoblasts to promote osteogenesis. BMPs (at least 20 species) belong to the transforming growth factor (TGF) *β* family, and as their name indicates, they are involved in bone metabolism as a component of bone matrix [78]. BMPs can cause ectopic bone formation when injected subcutaneously or intramuscularly [79]. Mutations in genes encoding BMPs in animals and humans lead to osteogenesis disorders, demonstrating the important role of these proteins in bone metabolism [80]. BMP-2, BMP-4, BMP-5, BMP-6, and BMP-7 are related to osteogenesis because of their ability to stimulate the expression of the transcription factors Runx2 and Osx [81]. In addition, BMP-2 can be specifically expressed in the cartilage assembly area, plays an important role in the proliferation and maturation of chondrocytes, and can enhance endochondral ossification [82]. According to Bandyopadhyay et al. [83], the lack of BMP-2 and BMP-4 in mice severely impairs osteogenesis, and mice who do not express BMP-2 in their limbs are prone to spontaneous fractures. Therefore, it is of great significance that the activation of the piezo1 protein induces the expression of BMP-2 to indirectly regulate the osteogenic effect of osteoblasts.

In subsequent studies, piezo1 was confirmed to be a real mechanical transducer that plays an important role in the development, growth, and maintenance of biological bones [84, 85]. In the experiment, the researchers suppressed the expression of piezo1 by simulating a microgravity environment and found that the function of osteoblasts was reduced. Similarly, some researchers [86, 87] have used specific siRNA transfection to silence the piezo1 gene to inhibit the expression of the piezo1 protein and have reached the same conclusion. In addition, researchers also found that piezo1 can mediate mechanical stimulation to induce Ca^2+^ influx to activate the CaMKII/Creb signaling pathway in osteoblasts to promote osteoblast differentiation [88, 89]. In subsequent studies, Zhou et al. [90] proposed that piezo1 is activated when the fluid shear stress is transferred to cause Ca^2+^ influx. They cooperate to activate NFATc1 and YAP1 and cascade transcription factors and induce dephosphorylation to promote the formation of the NFAT/YAP1 combined enzyme complex. This is a new mechanism to influence osteoblast differentiation. They also found that the loss of piezo1 in MSCs inhibits osteoblast differentiation, increases bone resorption, and causes multiple spontaneous fractures in newborn mice.

In the most recent studies, Sasaki et al. and Song et al. [91, 92] found that MC3T3-E1 osteoblasts need piezo1 to adapt to external mechanical fluid shear stress and partly induce the expression of the osteogenic Runx-2 gene through the AKT/GSK-3*β*/*β*-catenin pathway to achieve osteogenesis. Runx-2 belongs to the Runx transcription factor family, which also includes Runx-1 and Runx-3, and plays an important role in osteoblast differentiation. Runx-2 gene deletion leads to the complete loss of osteoblasts in mice [93, 94], and the mutation of Runx-2 in humans causes cleidocranial dysplasia (CCD), which is an autosomal dominant disease that causes significant abnormalities in bones due to intramembranous ossification [95]; these findings suggest that Runx-2 is the master gene for osteoblast differentiation [96, 97]. Regarding the specific effect of Runx-2, studies have shown that this transcription factor can upregulate the expression of osteoblast-related genes in osteoblasts [98]; therefore, this transcription factor plays an important role in the early development of osteoblasts. In addition, researchers [92] have also used small molecule agonists and inhibitors of piezo1 to study the effects on osteoblasts. The results have shown that inhibiting expression of piezo1in osteoblasts can significantly reduce the bone mass and strength of mice. In contrast, the use of Yoda1 agonists in adult mice can increase bone mass.

**Figure 5:**
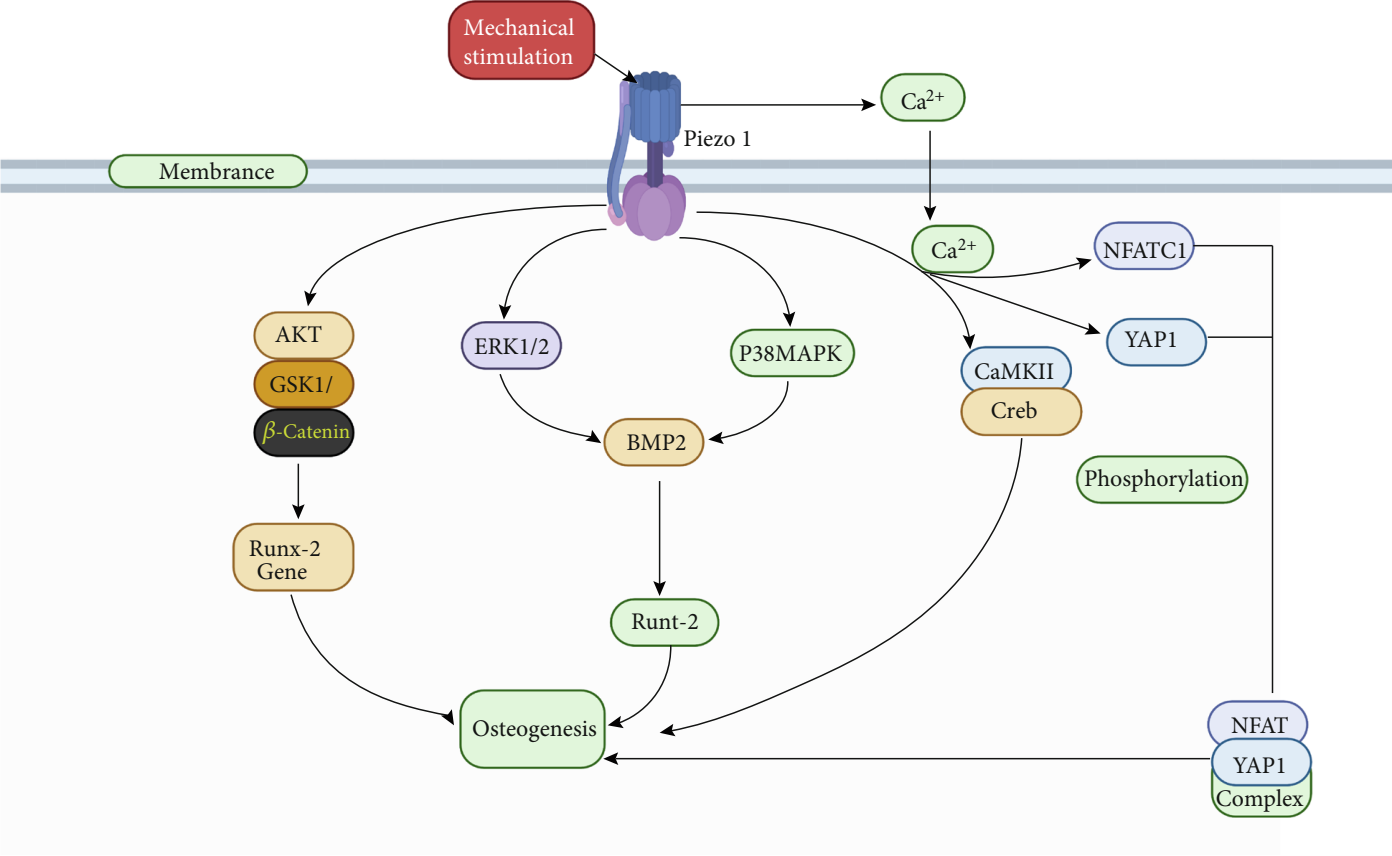
Modulation of piezo1 in osteoblasts.

Therefore, the abovementioned studies demonstrate that piezo1 is a mechanically sensitive ion channel through which osteoblasts can sense and respond to changes to influence their own osteogenic trends under external mechanical loads. Piezo1 can influence the osteogenesis of osteoblasts by regulating the expression of related factors or genes through certain signaling pathways and ultimately affect the degeneration of the human spine when it is activated (Figure 5).

### 2.3. Piezo1 Induces Osteoclast Differentiation to Achieve Osteodestructive Responses

Osteoclasts are specific multinucleated macrophages that are produced by the differentiation of monocytes/macrophage precursor cells on or near the bone surface [99]. Bone remodeling is the main metabolic process involved in regulating bone structure and function. Osteoclasts are the main participants in this process [100]. Bone homeostasis depends on the absorption of bone by osteoclasts and the formation of bone by osteoblasts [101]. An imbalance in this tight coupling process can lead to diseases such as osteoporosis [100, 102]. Bone resorption is a unique function of osteoclasts and a multistep process in which immature osteoclast precursors proliferate first and assume the osteoclast phenotype; then, mature osteoclasts degrade the organic and inorganic phases of bone [103]. To date, drugs, such as those for osteoporosis, have been developed that are aimed at inhibiting these cells [104, 105]. Osteoclasts are also regulated by a variety of cytokines including osteoprotegerin (OPG), nuclear factor receptor activator- (NF-) *κ*B (RANK), and RANK ligand (RANKL), which together regulate osteoclast function [106]. In addition, the mechanism of communication between osteoclasts and osteoblasts is critical to bone cell biology. Existing studies [107] have confirmed that osteoblasts and osteoclasts can communicate with each other through direct cell-cell contact, cytokines, and extracellular matrix interactions.

Jin et al. [108] evaluated the function of the piezo1 protein in the homeostasis of periodontal ligament tissue under a static mechanical load and reported for the first time that piezo1 mediates osteoclast differentiation. In their experiment, they found that the expression of piezo1 increased to varying degrees after human periodontal ligament cells were isolated, cultured, and pressurized for different periods of time. However, the formation of osteoclasts under mechanical stress in a pretreatment coculture system was inhibited when GsMTx4 was administered to inhibit piezo1. In addition, they also experimentally demonstrated that the NF-*κ*B signaling pathway is involved in inducing osteoclast production under mechanical stress, but the specific signal transduction mechanism has not been studied clearly. In further research, Wang et al. [109] used piezo1 knockout mice as experimental models and found that mice lacking the piezo1 gene in osteoblasts showed decreased bone mass and increased bone resorption after loading. However, the mice showed normal bone mass and bone resorption when the piezo1 gene in osteoclasts was knocked out and compared with the control group. They also elaborated on a new mechanism of interaction between osteoblasts and osteoclasts: piezo1 in osteoblasts controls the expression of type II and type IX collagen in response to external mechanical stimuli; in turn, these subtypes of collagen regulate the differentiation of osteoclasts. Furthermore, piezo1 mainly plays a role in osteoblasts and coordinates bone resorption of osteoclasts in a noncell-autonomous manner. In a recent study investigating the role of shear stress amplitude and stimulation time in the induction of osteoclast formation by hematopoietic progenitor cells, Bratengeier et al. [110] investigated the response of mouse hematopoietic progenitor cells to 2-minute dynamic fluid flow stimulation under precisely controlled fluid shear stress. In the experiment, they quantified the response of mouse hematopoietic progenitors by measuring the extracellular ATP concentration, cellular immunology of the piezo1 protein, Ca^2+^ concentration in the sarcoplasmic/endoplasmic reticulum and ability of ATPase 2 (SERCA2), and soluble factors produced by mechanically stimulated cells to regulate osteoclast differentiation. The results showed that a low stimulus amplitude corresponded to activation of the piezo1 channel and SERCA2, increased Ca^2+^ concentration in the sarcoplasmic/endoplasmic reticulum, decreased concentration of extracellular ATP, and inhibition of osteoclastogenesis and absorption area, while a high stimulus amplitude corresponded to bone destruction.

**Figure 6:**
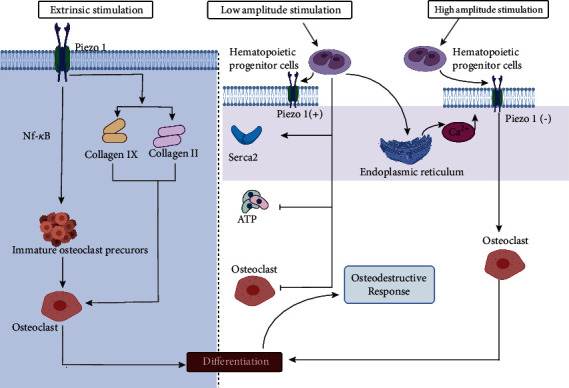
Piezo1 induces differentiation of osteoclasts to achieve osteodestructive responses.

Thus, piezo1 not only regulates the effects of osteoclasts by regulating the expression of bone matrix proteins including type II and IX collagen in osteoblasts but also is affected by the amplitude and duration of external mechanical stimulation to regulate osteoclast differentiation. Ultimately, piezo1 affects bone homeostasis and participates in the process of spinal degeneration (Figure 6).

### 2.4. Piezo1-Induced Apoptosis of Chondrocytes

The cartilaginous endplate located on the upper and lower sides of the intervertebral disc is one of the main structures of the intervertebral disc. Its structure is similar to that of articular cartilage, but it is not connected to the bony structure [111, 112]. As a transitional tissue between the upper and lower vertebral bodies, the cartilaginous endplate not only absorbs the mechanical pressure load of the spine to prevent bulging of the nucleus pulposus from impacting adjacent vertebral bodies but also acts as one of the important solute transport pathways for the nucleus pulposus (the cartilage endplate pathway) [113, 114]. The mature intervertebral disc is the largest organ without a blood supply in the human body. It needs to obtain a nutrient supply from the penetration of the cartilaginous endplate [115–117]. Therefore, maintaining the normal physiological shape and function of the intervertebral disc is essential for the health of the cartilaginous endplate. Among various unfavorable factors that accelerate cartilage endplate degeneration, such as gene mutations, apoptosis, and homeostatic damage, abnormal stress is one of the most important factors because it usually directly leads to damage to the cartilaginous endplate and surrounding tissues [118, 119]. As a mechanically sensitive protein channel, piezo1 plays an important role in the induction and mediation of abnormal stress and participates in the degeneration of the cartilage. Unfortunately, the current studies on chondrocyte degeneration caused by piezo1 mostly focus on the knee joint, and there is still a lack of studies on the signaling pathways related to the degeneration of the cartilaginous endplate. However, the phenotype of cells composing the cartilaginous endplate is generally considered to be chondrocytes [120, 121]. Some experiments have also used immunohistochemical methods to determine that the human thoracic cartilaginous endplate cells express type II collagen, which is consistent with the articular cartilage cells from different parts [122, 123]. Therefore, the effect of piezo1 on the degeneration of the cartilaginous endplate can be revealed by describing its effect on articular cartilage.

Lee et al. [124] first measured the presence and quantity of piezo1 in a mouse articular cartilage. Their experiments showed that piezo1 was strongly expressed in chondrocytes and with a high-level load; the ability of chondrocytes to obtain calcium ions increases significantly, and the apoptotic rate observably increased. However, significant calcium influx was not observed in the cartilage cells, and the apoptotic rate of chondrocytes was also greatly reduced after silencing piezo1 with specific siRNA; in addition, the use of the inhibitor GsMTx4 against piezo1 in the experiment also greatly reduced the apoptotic rate of chondrocytes, indicating that there is a mechanical conduction relationship between piezo1 and articular cartilage cells [125]. Similarly, when Yang et al. [126] studied the expression characteristics of piezo1 in a stress model of human degenerative chondrocytes, they found that piezo1 was expressed stably not only in mouse chondrocytes but also in human chondrocytes and was influenced in a time-dependent manner by mechanical stress. When studying the mechanism of ion action, Servin-Vences et al. [127] found that the piezo1 can mediate the intrachondral current induced by tension using a high-speed pressure clamp method. Their experiments also confirmed that mechanical stress can promote Ca^2+^ influx from the extracellular matrix into the chondrocytes through the piezo1 ion channel. Similarly, the study by Du et al. [128] also confirmed that Ca^2+^ in chondrocytes is essential for the transduction of stretch stimulation signals. Mechanically sensitive ion channels including TRPV4, piezo1, and piezo2 play different roles in the process of calcium oscillations caused by stretch stimuli of different intensities [129, 130].

Therefore, piezo1 is expressed in mammalian chondrocytes including humans' chondrocytes, which is a necessary condition for causing calcium influx in chondrocytes after mechanical stimulation [128]. Overload of Ca^2+^ activates intracellular messengers and regulates the kinase cascade to mediate chondrocyte apoptosis and is the key mechanism of chondrocyte apoptosis [131]. When Li et al. [132] studied the pathway by which piezo1 mediates chondrocyte apoptosis, the activation of piezo1 was found to upregulate the expression of Bax (a proapoptotic protein) and caspase-3 (an effector protein that can degrade intracellular structure) and inhibits the expression of the anti-apoptotic protein Bcl-2. A caspase is a general term for a cysteine protease involved in cell apoptosis [133, 134] that can transmit apoptotic signals, such as abnormal mechanical tension and inflammation, to proteolytic cascade reactions to lyse and activate other caspases and then degrade intracellular targets, finally leading to cell apoptosis [135, 136]. In the case of piezo1, which mediates chondrocyte apoptosis, the specific mechanism is that piezo1 activates the downstream classic MAPK/ERK 1/2 signaling pathway when activated by mechanical stress. Then, mechanical signals are transmitted to the cell nucleus directly through the ERK1/2 pathway, causing the corresponding changes in the relevant apoptotic genes, such as Bcl-2, Bax, and caspase-3 in the nucleus, and finally leading to cell apoptosis [131]. Similarly, other studies [137, 138] have confirmed that piezo1 can also initiate cell apoptosis through the MAPK/ERK5 signaling pathway and endoplasmic reticulum stress with calcium ions as the second messenger. Piezo1 is involved in the late apoptosis of chondrocytes in patients with osteoarthritis. Studies have also proposed that this protein is a potential therapeutic target for inhibiting chondrocyte apoptosis.

**Figure 7:**
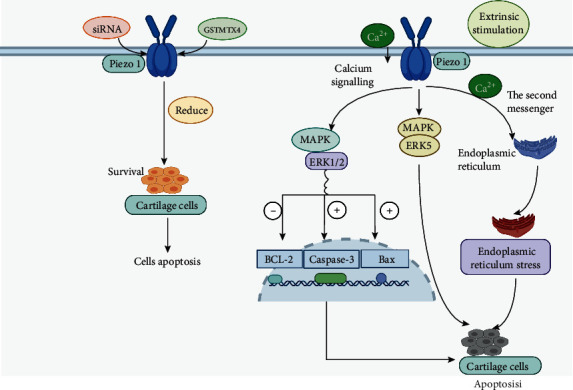
Piezo1 induces apoptosis of chondrocytes

In summary, piezo1 can induce chondrocyte and cartilaginous endplate cell apoptosis through different signaling pathways and participate in joint and intervertebral disc degeneration with external mechanical stimulation. The specific pathway of action is shown in Figure 7.

### 2.5. Piezo1 Mediates Inflammation and Apoptosis in Nucleus Pulposus Cells

The nucleus pulposus is the gel-like part in the center of the intervertebral disc that is located in the posterior position and accounts for 50% to 60% of the cross-sectional area of the intervertebral disc [139, 140]. It is in close contact with the cartilage endplate and is the main way for the intervertebral disc to receive nutrition through the cartilage endplate and the main part involved in nutrient osmotic exchange. It is composed of water (70-90%), nucleus pulposus cells, proteoglycans, and type II collagen [141]. The proteoglycans include the larger aggrecan, which is responsible for retaining water within the nucleus pulposus [142, 143]. In addition, it provides versican, which binds to hyaluronic acid. This hydrophilic matrix is responsible for maintaining the height of the intervertebral disc [144]. It is this unique composite material that makes the nucleus pulposus elastic and flexible to absorb pressure under compression [145]. The nucleus pulposus together with the cartilage endplates of the upper and lower vertebral bodies and the surrounding fibrous annulus build a closed buffer system to resist gravity and tension. When bearing an external force, the nucleus pulposus evenly transfers the force to the surrounding fibrous annulus and the vertical cartilage endplate, avoiding a certain part of the intervertebral disc from being damaged due to excessive load; it also has the effect of balancing stress. When the spine moves, the nucleus pulposus acts as a fulcrum similar to a ball bearing, assisting other parts of the spine to complete physiological activities. The spheroidal structure of the nucleus pulposus in the backward position is of great significance for dispersing pressure and supporting movement with large angles and high frequency [146–148]. Although there are many factors that cause apoptosis of nucleus pulposus cells, the role of external improper mechanical stress is still the main factor [149, 150]. Apoptosis of nucleus pulposus cells for any reason will cause the “closed buffer system” to lose balance and reduce the effect of balancing pressure, leading to decreased function of the intervertebral disc and eventually degenerative disease of the intervertebral disc. Whether piezo1, a sensitive channel that mediates mechanical stimulation, is important in inducing apoptosis in nucleus pulposus cells is worth investigating.

Yang et al. [151] used the multichannel cell stretch stress-loading system FX-4000T to treat chondrocytes. A loading frequency of 0.5 Hz and a cell elongation of 20% were loaded. According to the cell processing time, the cells were divided into 0 h, 2 h, 12 h, 24 h, and 48 h mechanical stress groups. RT-PCR and Western blot were used to evaluate the expression of the piezo1, showing that it was extensively expressed in the cytoplasm and nucleus of the nucleus pulposus cells. With an increased stress-processing time, the fluorescence intensity of the protein also increased. Similarly, researchers [152] collected specimens that were surgically removed due to lumbar degenerative diseases as experimental samples. Samples from a total of 26 patients (15 males and 11 females) were collected, including 3 cases of Pfirrmann II degeneration, 8 cases of Pfirrmann III degeneration, and 15 cases of Pfirrmann IV degeneration. According to the degree of degeneration, the tissue specimens with Pfirrmann II degeneration were used as the control group, and those with Pfirrmann III and IV degeneration were used as the degeneration group. The localization and expression level of the piezo1 protein in tissues with different degrees of degeneration were detected by immunohistochemistry. The results confirmed that the piezo1 protein was expressed in the nucleus pulposus cells of the intervertebral disc with different degrees of degeneration. The results also showed that the piezo1 protein is differentially expressed in intervertebral disc tissues with different degrees of degeneration, and its expression level is related to the degree of degeneration. Finally, a hypothesis was proposed by them that the piezo1, a mechanosensitive ion channel protein, might be involved in the degeneration of the nucleus pulposus cells in the intervertebral disc.

In further research, Yang et al. [153] interfered with the expression of the piezo1 protein by transfecting an shRNA-piezo1 vector into nucleus pulposus cells; they measured the cytoplasmic Ca^2+^ concentration, change in mitochondrial membrane potential, and mRNA and protein levels of piezo1 in the cells to study the effect of piezo1. The results showed that the cytoplasmic Ca^2+^ concentration and conversion rate of mitochondrial membrane potential in cells interfered with shRNA were reduced. shRNA-piezo1 was found to protect nucleus pulposus cells by reducing the intracellular Ca^2+^ level and changing the mitochondrial membrane potential. Li et al. [154] also proposed that the piezo1 protein may play a key role about the apoptosis of human nucleus pulposus cells through mitochondrial dysfunction and endoplasmic reticulum stress under abnormal load conditions.

**Figure 8:**
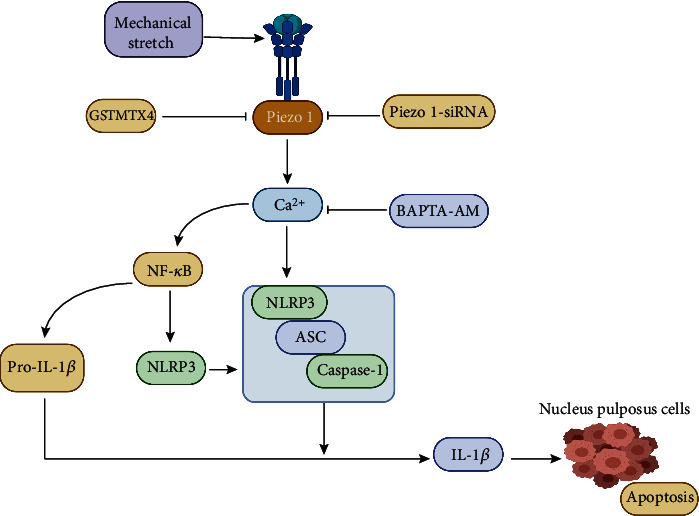
Piezo1 mediates apoptosis of the nucleus pulposus.

The abovementioned studies all suggest the relevance of the piezo1 protein in the apoptosis of nucleus pulposus cells, but there have been no reports on how piezo1 mediates the specific signaling pathway of apoptosis in nucleus pulposus cells. However, recently, when Sun et al. [155] studied an inflammation model of nucleus pulposus cells mediated by piezo1, they linked the inflammation mediated by piezo1 in nucleus pulposus cells to PYD domains-containing protein 3 (NLRP3). The excessive activation of the NLRP3 inflammasome is known to result in overproduction of downstream IL-1*β*, which participates in the pathogenesis of human intervertebral disc degeneration [156–158]. Their study confirmed that activation of piezo1 after mechanical stretching induced activation of caspase-1 and increased production of IL-1*β*, which can promote the assembly of NLRP3. In addition, the Ca^2+^/NF-*κ*B pathway was inhibited by them with transfection of specific siRNA, which reduced the activity of the piezo1-dependent NLRP3 inflammasome. They concluded that the expression of piezo1 and the NLRP3 inflammasome increased in a time-dependent manner and that a specific mechanism of apoptosis of nucleus pulposus cells that activated piezo1 increased the intracellular calcium load and upregulated the expression of NLRP3 by activating the NF-*κ*B pathway to mediate inflammation and apoptosis of nucleus pulposus cells. In addition, piezo1 can also be used as a second stimulus to directly promote the assembly of NLRP3, activation of caspase-1, and production of IL-1*β* to mediate the inflammatory response and apoptosis of nucleus pulposus cells even in the absence of mechanical stimulation [159–161]. Therefore, piezo1 is not only stably expressed in human nucleus pulposus cells but also mediates inflammation and apoptosis of nucleus pulposus cells through a certain mechanism. It plays an important role in the occurrence and development of intervertebral disc degeneration according the abovementioned studies (Figure 8).

## 3. Piezo1 and Other Human Diseases

The function of piezo1 is involved in a variety of biological diseases. Piezo1 deficiency causes changes in osmotic pressure in red blood cells and ultimately leads to anemia [162–165]. Many studies have shown that the piezo1 protein is expressed in the endothelial cells of mice during vascular development, and the loss of the piezo1 gene can lead to insufficient orientation of stress fibers and cells in response to shear stress. Embryos with a deleted piezo1 gene have defects in vascular remodeling that can lead to death in the second trimester [166, 167]. In addition, piezo1 is also highly expressed in the smooth muscle cells of small arteries and plays an important role in the regulation of myogenic arterioles [168, 169]. Piezo1 affects the diameter and wall thickness of arterioles in hypertensive patients and participates in the remodeling of arterioles. Piezo1 mediates the depolarization of vascular endothelial cells to connect them to smooth muscle cells [45, 170] and then triggers communication with mesenteric vascular endothelial cells through gap junctions, resulting in vasoconstriction [171–173]. Thus, piezo1 is important in the mechanical biology of the blood vessels and in related clinical diseases, such as atherosclerosis and hypertension [174–176].

Piezo1 is a sensor that controls the development and maintenance of lymphatic valves in the signal transmission pathway of mechanical force; it also participates in the formation of lymph [177–179]. Human piezo1 gene mutations or loss of function mutations can lead to autosomal recessive congenital lymphatic dysplasia, which is related to congenital lymphedema with pleural effusion [180–182].

Romac et al. [183] experimentally confirmed that piezo1 can mediate pressure-induced pancreatitis. Mechanical pressure can activate the piezo1 protein channel on the membranes of pancreatic acinar cells and other parts of the pancreas, allowing Ca^2+^ to flow into the cell to increase the Ca^2+^ concentration; these high concentrations of Ca^2+^ induce protease activation and ultimately lead to pancreatitis. In further research, a recent study by Swain et al. [184, 185] showed that when mechanically stimulating pancreatic acinar cells, calcium ion permeation through an activated piezo1 protein channel is the first step in stress-induced pancreatitis, and piezo1-induced TRPV4 channel opening is the main factor leading to pancreatitis.

Piezo1 is closely related to a variety of human tumors, such as synovial sarcoma. Piezo1 is a potential regulator of synovial sarcoma cell viability and may play a role in invasion and metastasis proliferation [186]. Li et al. [187] studied the relationship between breast cancer and piezo1 and found that when a patient's piezo1 mRNA level increases, the overall survival rate is significantly reduced, revealing the role of piezo1 in breast cancer progression. Similarly, piezo1 is also involved in the expansion and metastasis of colon cancer [188], stomach cancer [175, 189, 190], glioma [191] , bladder carcinoma [192], and lung cancer cells [193]. Overexpression of piezo1 has an adverse effect on the prognosis of glioma patients and can be used as a prognostic factor for glioma [194, 195]. This may be a new prognostic indicator for glioma patients. The function of piezo1 ion channels in human osteosarcoma cells is also related to apoptosis, invasion, and cell proliferation [196, 197].

In conclusion, piezo1 is clearly widely expressed in multiple tissues and cells of the human body and is involved in the occurrence of various human diseases (Table 1). The role piezo1 plays in the pathogenesis of diseases will be gradually discovered, and new targets and ideas will be provided for the treatment of these diseases.

## 4. Conclusions and Prospects

### 4.1. Conclusions

Spinal degeneration is a common clinical disease. As a chronic disease, its clinical manifestations, such as long-term low back pain, not only affect the life and work of patients but also cause heavy economic burdens to patients, their families, and society [198–200]. Spinal degeneration includes the degeneration of the vertebral bodies and intervertebral discs, and disc degeneration is a common and important form of degeneration. The intervertebral disc is composed of the central nucleus pulposus, the outer fibrous annulus, and the upper and lower cartilage endplates, which link the upper and lower vertebral bodies, bear mechanical loads such as compression, extension, flexion and torsion, and play an important role in bearing body weight and buffering pressure loads [149]. Lotz et al. [150] showed that the magnitude and duration of pressure are positively correlated with the rate of intervertebral disc cell apoptosis, which is an important factor for leading to intervertebral disc degeneration and herniation. Therefore, the study of the biomechanical signal transduction mechanism of human spinal cells has become an important direction for studying the mechanism of spinal degeneration.

Mechanosensitive ion channels are a type of ion channels that can sense changes in the mechanical stress of the cell membrane and quickly convert the sensed mechanical signals into electrical or chemical signals to regulate the life activities of the cells. Piezo1 is a new type of mechanically sensitive ion channel discovered by Coste et al. in 2010 [1, 21]. As a member of the mechanically sensitive ion channel family, it is closely related to the induction and conduction of mechanical signals in biomechanics. It has been confirmed that piezo1 is expressed in a variety of cells, such as gastric antrum G cells, skin, bladder, kidney, lung, endothelial cells, red blood cells, and root ligament cells, according to existing studies [51]. Moreover, piezo1 is also expressed in MSCs, osteoblasts, osteoclasts, chondrocytes, and nucleus pulposus cells [83, 108, 126, 129, 153] and is involved in mediating their differentiation and apoptosis, resulting in decreased bone density and function of intervertebral disc. In addition, piezo1 is involved in the pathological progression of bone metabolic diseases, degenerative arthritis and other orthopedic diseases (Table 2).

In MSCs, the expression of piezo1 can promote expression of BMP2, which induces MSCs to differentiate into osteoblasts while inhibiting their differentiation into adipocytes. Piezo1 can also induce the release of ATP and regulate the migration and transformation of MSCs by activating the downstream PYK2 and MEK/ERK signaling pathways after receiving the signal from the purinergic P2 receptor to affect the hardness and density of the vertebral body [129, 138].

The differentiation of osteoblasts is affected by piezo1 in four ways: (1) the expression of piezo1 induces the expression of BMP-2 through the ERK1/2 and p38MAPK signaling pathways. Then, BMP-2 induces the expression of Runt-2 in osteoblasts to promote osteogenesis [83]. (2) Piezo1 mediates Ca^2+^ influx induced by mechanical stimulation and then activates the Ca/MKII/Creb signaling pathway in osteoblasts to promote osteogenic [85]. (3) Piezo1 induces the expression of the osteogenic gene Runx-2 through the AKT/GSK-3*β*/*β*-catenin pathway to promote osteoblast differentiation to achieve osteogenic effects [85]. (4) The activation of the piezo1 protein channel causes Ca^2+^ influx, which synergistically activates NFATc1, YAP1, and cascade transcription factors, inducing their dephosphorylation to promote the formation of NFAT/YAP1 combined enzyme complexes to affect osteoblast differentiation [86, 87].

Piezo1 regulates differentiation of osteoclasts by regulating the expression of BMPs including collagens 2 and 9 [108]. In addition, it influences the production of osteoclasts induced by mechanical stress through the NF-*κ*B signaling pathway to affect bone homeostasis [109].

Piezo1 can mediate apoptosis of chondrocytes by activating the downstream MAPK/ERK5 signaling pathway and the classic MAPK/ERK 1/2 signaling pathway [126, 127]. In the classic MAPK/ERK 1/2 pathway, ERK1/2 can directly transmit mechanical signals to the nucleus to cause the response of apoptosis-related genes such as Bcl-2, Bax, and caspase-3 to lead to apoptosis [128, 129]. In addition, by regulating Ca^2+^ influx, piezo1 can also cause endoplasmic reticulum stress and mitochondrial disorders to induce chondrocyte apoptosis [130, 131].

Piezo1 also plays a key role in apoptosis of nucleus pulposus cells through inducing mitochondrial dysfunction and endoplasmic reticulum stress pathways as in chondrocytes [153]. The intracellular calcium load increases when the piezo1 protein channel is activated, which upregulates the expression of NLRP3 by activating the NF-*κ*B pathway to mediate inflammation and apoptosis of nucleus pulposus cells [154]. In addition, piezo1 can also be used as a second stimulus to directly promote the assembly of NLRP3, activation of caspase-1, and production of IL-1*β* to mediate the inflammatory response and apoptosis of nucleus pulposus cells [155].

### 4.2. Prospects

Piezo1 is a newly discovered channel protein in recent years [1, 21]. To date, despite the growing number and depth of studies on piezo1, there are relatively few limited studies on piezo1 in spinal degenerative diseases. The details are as follows: (1) the specific mechanism of the classical signaling pathway of piezo1 in osteoclasts and nucleus pulposus cells is unclear and not detailed. (2) The degeneration of the cartilage endplate, which leads to barriers of transport of metabolites and nutrients in the intervertebral disc, is one of the important initiating factors for intervertebral disc degeneration. Although existing studies have shown that the phenotype of the cartilage endplate cells of the intervertebral disc is the same as that of cells in other articular cartilage, there is a lack of literature about piezo1 in the cartilage endplate directly relating to how the piezo1 mediates signals to induce apoptosis of cartilage endplate cells under mechanical stress. (3) The annulus fibrosus is mainly composed of type I collagen fibers, which surround the nucleus pulposus through spirally arranged fibrous tissue and attach to the vertebral body [201, 202]. This unique structure gives the annulus fibrosus the ability to withstand loads and limit excessive spinal torsion, rotation and bending [203–205]. Its structural integrity is essential for limiting the protrusion of the nucleus pulposus and maintaining the physiological internal pressure of the intervertebral disc under load, and it plays a vital role in the biomechanical properties of degeneration of the intervertebral disc [206]. As one of the important structures maintaining the integrity of the intervertebral disc, the state of the annulus fibrosus is influenced by many factors. An inappropriate external stress stimulus is still the main factor affecting the annulus fibrosus and leading to its rupture, which ultimately affects the function of the intervertebral disc. Therefore, whether piezo1 is involved in the pathological process of rupture and apoptosis of annulus fibrosus cells when mediating external mechanical stimuli through signaling pathways, similar to what occurs in chondrocytes and nucleus pulposus cells, and ultimately causing nucleus pulposus tissue to protrude and compress the nerve root and spinal cord remains unknown. Unfortunately, there are no reports about piezo1 in annulus fibroblasts or tissues, and it is unknown whether piezo1 is even expressed in annulus fibroblasts or tissues. Therefore, this can also become a new research direction regarding intervertebral disc degeneration. (4) When mechanical stimulation activates the piezo1 protein channel, the influx of Ca^2+^ occurs. Patients with spinal degeneration often also have accompanying hyperplasia of the vertebral body and calcification of the anterior and posterior longitudinal ligaments. Is this related to the overexpression of piezo1 to lead to an increased intracellular calcium load? If piezo1 is involved in each of these diseases, then the pathogenesis is worth investigating.

Unfortunately, there are no relevant studies or experimental reports about the abovementioned discussion. Hopefully, this article will provide some directions for further research about the role of piezo1 in spinal degenerative disease. The mechanism of action of piezo1 in spinal degenerative diseases should be clearly studied with deepening research, and piezo1 may become a new factor for the prevention and treatment of spinal degenerative disease in the imminent future.

## Figures and Tables

**Table 1:** Actions of piezo1 in other human diseases.

Disease types	Action	References
Anemia	Changes cell osmotic pressure	[162–165]
Hypertension	Regulates arteriole smooth muscle	[168, 169]
Atherosclerosis	Promotes atherosclerosis	[174–176]
Congenital lymphedema	Absence of piezo1 leads to lymph dysplasia	[178–180]
Pancreatitis	Induces Ca^2+^ expression	[183–185]
Colon cancer	Promotes expansion and metastasis	[188]
Gastric cancer	Promotes expansion and metastasis	[173, 189]
Breast cancer	Enhanced proliferation	[187]
Synovial sarcomas	Increased proliferation	[186]
Osteosarcoma	Inhibits apoptosis and promotes invasion and proliferation	[196, 197]
Bladder carcinoma	Promotes expansion and metastasis	[192]
Lung cancer	Promotes migration and tumor growth	[193]
Gliomas	Increased proliferation	[194, 195]

**Table 2:** Actions of piezo1 on cells of the vertebral body and intervertebral disc.

Cell type	Action	References
MSCs	Regulates the differentiation	[61, 65]
Osteoblasts	Regulates osteogenesis of osteoblasts	[83–87]
Osteoclasts	Induces differentiation to achieve the Osteoclast effect	[108, 111]
Chondrocytes	Induced chondrocyte apoptosis	[126–131]
Nucleus pulposus cells	Mediates inflammation and apoptosis	[153–155]
Annulus fibrosus	Unclear	
